# The Impact of Immunosenescence on Pulmonary Disease

**DOI:** 10.1155/2015/692546

**Published:** 2015-06-23

**Authors:** Michelle A. Murray, Sanjay H. Chotirmall

**Affiliations:** ^1^Department of Respiratory Medicine, Mater Misericordiae Hospital, Eccles Street, Dublin 7, Ireland; ^2^Lee Kong Chian School of Medicine, Nanyang Technological University, Singapore 308232

## Abstract

The global population is aging with significant gains in life expectancy particularly in the developed world. Consequently, greater focus on understanding the processes that underlie physiological aging has occurred. Key facets of advancing age include genomic instability, telomere shortening, epigenetic changes, and declines in immune function termed immunosenescence. Immunosenescence and its associated chronic low grade systemic “inflamm-aging” contribute to the development and progression of pulmonary disease in older individuals. These physiological processes predispose to pulmonary infection and confer specific and unique clinical phenotypes observed in chronic respiratory disease including late-onset asthma, chronic obstructive pulmonary disease, and pulmonary fibrosis. Emerging concepts of the gut and airway microbiome further complicate the interrelationship between host and microorganism particularly from an immunological perspective and especially so in the setting of immunosenescence. This review focuses on our current understanding of the aging process, immunosenescence, and how it can potentially impact on various pulmonary diseases and the human microbiome.

## 1. Introduction

Global aging of the human race, particularly in the developed world, is becoming a key factor in the development and progression of pathological disease. Morbidity and mortality from pulmonary illness have interestingly increased while those from other prevalent diseases such as cardiovascular or neurological have remained stable or in some cases decreased. This has led to recognition of the importance of age-related changes to the development and progression of lung disease. While a multitude of cellular and molecular changes occur with age, their specific impact on the respiratory system, pulmonary physiology, and disease susceptibility remains undetermined. Establishing causation between these areas is a key first step to promoting greater understanding and improved research in the field. Age-related declines in immune function, termed “immunosenescence,” likely play a critical role in the manifestation of age-related pulmonary diseases such as infection, asthma, and chronic obstructive pulmonary disease (COPD). Coupled with the advent of emerging molecular detection techniques and genome-related information including epigenetic, transcriptomic, and proteomic data, determining the complex interrelation between biological aging, abnormal pulmonary function, and predisposition to lung disease in older individuals remains necessary to permit improved and focused therapeutics for this specialised cohort. This review aims to outline our current understanding of the process of aging, immunosenescence, and how these processes impact on the development and progression of pulmonary disease.

## 2. Aging and the Population

Over the last decade, the proportion of the developed world's population over the age of 65 years has increased by more than 10%. Furthermore, it is projected to increase further to over 20% by 2030 [1]. In conjunction with an aging population, life expectancy continues to increase globally and is expected to reach the mid-70s by 2050 [2]. Pulmonary morbidity and mortality have concurrently increased as the population has aged conferring increased risks of infection, COPD, and asthma [3–7].

Aging is described as encompassing biological, cellular, molecular, and subcellular components, all integral to normal immune function and immunosenescence with advancing age. Biological aging includes diminishment of physiological integrity consequently impairing organ function and increasing frailty [8]. All such manifestations are allied to acquisition of molecular damage from environmental and metabolic sources that subsequently lead to disease susceptibility and eventual death. Key features of mammalian aging include genomic instability, telomere shortening, extracellular matrix alteration, epigenetic changes, modified cell communication, and dysregulated immune function [9]. Such age-related phenomena impact on the genome, transcriptome, proteome, and metabolome which in turn dictate biological phenotypes observed in pulmonary health and disease. A pressing need to detect causal associations between the cellular and molecular manifestations of aging and lung disease suggests that an understanding of immunosenescence in the context of pulmonary health and disease is an important challenge for future research in the field [10].

## 3. Immunosenescence

Global aging has health implications [11]. The elderly suffer from more frequent and more severe community-acquired and nosocomial infections compared to younger individuals and tend to have poorer outcomes [12]. The clinical presentation is additionally often atypical creating diagnostic difficulties for clinicians. This is intrinsically linked to the physiological aging process and immune function. The immune system of older individuals declines with advancing age, increasing susceptibility to infection and cancer and also reduced vaccine responses [13]. Such physiological declines in immune function are termed “immunosenescence” and while aging is central to the process, other factors contribute to normal immune homeostasis and as such it can be a highly variable process between individuals. Immunosenescence is therefore defined as the impairment in both cellular and adaptive immunity as a result of age-related change [14] and in this paper we focus on its potential impact on a variety of pulmonary diseases.

Immunosenescence causes age-related declines in immune function at both cellular and serologic levels [7, 15]. Specific responses to foreign and self-antigens ensue promoting an increased susceptibility of the elderly to diseases including infection, cancer, autoimmune, and other chronic processes in addition to a poorer vaccine response. Both innate and adaptive arms of immune function are affected [16]. Autoimmunity, immunodeficiency, and immune-dysregulation are some of the theories put forward to account for this physiological phenomenon; however it is likely that a combination of these takes place* in vivo*.

Aging is associated with a chronic low grade inflammatory state [17]. As such, proinflammatory cytokines including TNF-*α*, IL-1, and IL-6 are systemically elevated. Such “inflamm-aging” may be part of the aging process itself; however it has been proposed in the pathogenesis of several age-related inflammatory diseases including atherosclerosis, diabetes, and Alzheimer's [18, 19]. Importantly, it has been observed that certain individuals age in a “healthy” manner without major health concerns. In this setting, genetic and environmental influences have a role; however it is known that the proinflammatory state in “healthy aging” is inhibited by cytokines such as IL-10 [16]. Oxidative stress also plays a key role in the immunosenescence process as it impacts on both innate and adaptive immunity despite the lack of clear mechanistic data. What is known however is that oxidative stress remains a major factor in accelerated aging models, due to an increased rate of telomere shortening consequent to DNA damage [20]. Therefore, targeting macrophages, granulocytes, and dendritic cells with antioxidants in murine models has shown some improvement through mechanisms including chemotaxis, IL-2 production, and natural killer (NK) cell activity; however human study remains lacking [21]. Targeting ways to reduce oxidative stress and thus improve immune function in the setting of immunosenescence represents a potential key area for future interest.

### 3.1. Immunosenescence and Inflammation

Immunity and inflammation are interlinked. The innate immune response includes macrophages, NK cells, and neutrophils, all providing a first-line defense against pathogens. Interestingly, while the function of these cells declines with age, their production actually increases. In the elderly macrophages have a reduced ability to secrete tumour necrosis factor (TNF), a key inflammatory cytokine, while IL-7 production by bone marrow stromal cells is also impaired [22, 23]. IL-7 is an essential cytokine for developing lymphocytes [24].

Pattern-recognition receptors such as the toll like receptors (TLRs) are utilized by the innate immune system to recognise specific molecular patterns present on pathogenic surfaces. TLRs are expressed on a variety of cells including macrophages, lymphocytes, and bronchial epithelia. Once engaged, TLRs stimulate the secretion of antimicrobial peptides and trigger an inflammatory response through cytokine and chemokine secretion to eliminate the offending pathogen. Studies in human and animal models have shown that TLR expression and function declines with age, resulting in a diminished production of inflammatory cytokines and a blunted inflammatory response that also results in dysregulation of the adaptive immune system through molecular cross talk [11].

There are also significant changes in humoral immune function in the elderly. These changes are characterised by decreased antibody responses and a reduced production of high-affinity antibodies. B-cell proliferation declines in aged mice due to declining B-cell activation and defective surface Ig/B-cell receptor affinity and signalling [25]. There is also loss of naive B-cells and an increase in memory cells with age [26], reducing the ability as one grows older to respond to novel antigens. Memory cells produced early in life however remain normal [27]. Aging is associated with shifts from Th1 to the Th2 cytokine profiles in response to immune stimulation. Overproduction of Th2 cytokines in this setting may in fact augment B-cell-mediated autoimmune disorders [28].

Finally, a reduction in cell-mediated immunity forms part of the aging immune profile. The thymus involutes and consequently native T-cells are reduced in both the blood and peripheral tissues of elderly individuals. There is an increase in memory cells particularly CD4+, CD8+, and regulatory T-cells. As a result, a shift in the ratio of naive to memory T-cells ensues in the periphery to maintain peripheral T-cell homeostasis. An improved basic understanding of immune dysfunction during the human aging process will likely increase possibilities of identifying achievable means to improve and potentially restore immune function consequently alleviating the burden of infectious and other pulmonary diseases in later life that currently carry significant morbidity and mortality [28].

## 4. Immunosenescence and Pulmonary Disease

### 4.1. Asthma and Allergy

While the asthmatic phenotype in children is well defined, “late-onset” asthma has lagged behind. This is largely explained by the heterogeneous nature of disease despite the similar treatment approaches. Until recently, phenotypes of “late-onset asthma” were based on aetiology, for instance, aspirin sensitivity, toxic exposures, or occupational influence or alternatively clinical disease characteristics such as mild, moderate, or severe. Terms such as brittle, near fatal, steroid resistant, asthma-COPD overlap syndrome (ACOS), or fixed airflow obstruction have also been utilized as descriptors for late-onset disease. Recent work however compared patients with late-onset mild-moderate disease to those with more severe disease. Interestingly, it was described that those with more severe disease were likelier to have nasal polyposis, fixed airflow obstruction, sputum eosinophilia, and higher serum neutrophilia but were less likely to be atopic suggestive of different underlying mechanisms that lead to late-onset disease [29, 30].

Consequently, mechanisms associated with late-onset asthma are incompletely understood. Suggestions are that it may occur as a consequence to viral infection that promotes persistent inflammatory change when coupled to the effects of immunosenescence [6, 7]. While asthma follows a Th2 cytokine bias in early life, studies from older asthmatics have indicated a possible role for a Th1 response in neutrophilic-predominant asthma, providing potential evidence of age-associated changes in the inflammatory and immune milieu [31]. Studies using murine models show diminished B-cell populations and transition from naive B-cells to antigen-expressing B-cell cohorts [32]. Reduction of antibody production may be responsible for the enhanced antigen persistence and specificity observed in the elderly. Thymic involution further causes T-cell population shifts, altered B-cell antigen processing, and eosinophil function coupled to reduction in phagocytic capability, all of which contribute to a unique immunological environment present in the elderly asthmatic [33]. Additionally, T-cells are highly activated in the elderly, with increased expression of human leukocyte antigen- (HLA-) DR and CD69. An increase in airway neutrophils has also been observed in older asthma subjects prompting suggestions of a differing asthmatic phenotype as one ages [34]. One of the major difficulties faced in diagnosing asthma in the older patient is the overlap of other symptoms that occur with other chronic age-related diseases including ischaemic heart disease, cardiac failure, and COPD [6]. Moreover, older adults will physiologically lose lung capacity over time and patients may develop a fixed obstructive defect on their pulmonary function tests making their interpretation and diagnosing asthma challenging in this age group [6, 7].

To assist in providing further evidence of airway inflammation in the elderly, measurement of exhaled nitric oxide (eNO) may be used as it is noninvasive however less reliable in older patients. In a study evaluating *n* = 2200 subjects, aged 25 to 75, it was found that an increased eNO was associated with advancing age [35]. This increase may be reflective of the altered distribution or activity of inflammatory cells within the airway irrespective of the presence of asthma. Older asthmatic subjects with evidence of atopy continue to exhibit airway eosinophil levels comparable to younger asthmatics, a scenario where eNO may be of value; however atopy remains less likely to occur with advancing age [34]. In late-onset asthmatics, the most notable difference in the airway cellular distribution is the increase in neutrophil abundance with a corresponding decrease in macrophages, seen to a lesser extent in older individuals without asthma [34, 36, 37].

The prevalence of allergic disease in the elderly has been estimated at between 5 and 10% [38]. Most often, the disease begins in childhood persisting throughout life and into older age. For some, allergy appears for the first time as an older adult. Changes in the immune system as previously described do contribute however, concurrent molecular changes occurring with age influence functional structures which have roles in aiding immune function [39]. With age, zinc transporters become less efficient at releasing zinc, leading to lower intracellular zinc concentrations that in turn impact immune efficiency [40]. Calcitriol, the active form of vitamin D, influences innate and adaptive immunity by acting upon antigen presenting cells and regulatory T-cells to attenuate the inflammatory response [36, 41]. Serum IgE concentrations dramatically decrease in the elderly explaining at least partially the absence of allergic asthmatic features; however interestingly the immunosenescence process does not impact directly on IgE concentrations and therefore atopic elderly individuals continue to demonstrate elevated serum IgE levels [42].

Th17 are a subgroup of T-cells that secrete IL-17A, IL-17F, TNF-*α*, and IL-22 [43]. Several murine models of allergic asthma suggest that both IL-17A and IL-17F have key roles in the regulation of inflammation within the airway. This promotes neutrophil recruitment [44, 45], enhances Th2 driven eosinophilia [46], and increases MUC5A expression [47]. IL-17A and IL-17F are detected in the asthmatic airway [48]. Th17 cells are maintained by IL-23 and this axis is important in the host response to bacterial and viral infection and additionally may have a role in infection within the context of “late-onset asthma” [49]. Recent studies agree that an increased IL-17 expression occurs with aging while murine work suggests that infection with HSV2 [50] and* Brucella abortus* [51] in older mice causes higher levels of IL-17 to be produced compared to the younger mice who displayed primarily Th1 expression. These data do suggest a potential link between immunosenescence and asthma; however this relationship has not yet been clearly established. With limited longitudinal data available, further research in this field is clearly required to better understand and potentially therapeutically manipulate these complex relationships.

### 4.2. Pulmonary Infection

Respiratory infections remain a leading cause of morbidity and mortality worldwide especially in older adults. The increased risk of community-acquired pneumonia in elderly patients ranges from 15 to 30% independent of socioeconomic status or comorbidities [52]. Aspiration pneumonia in the elderly additionally accounts for up to 15% of pulmonary infection [53].* Streptococcal pneumonia* is the most common infectious cause of pneumonia following a viral illness worldwide [54]. The detection methods available to identify colonization by this organism remain poor by oropharyngeal swab and conventional culture which reveal only 6% of cases [55]. Molecular based methodologies can increase detection rates by up to 37% [56]. Despite advances in molecular based detection techniques, there is limited evidence addressing specific mechanisms by which immunosenescence predisposes to pneumococcal associated disease [57].

In addition to* Streptococcus pneumoniae*,* Neisseria meningitides*,* Haemophilus influenzae*,* Moraxella catarrhalis*, and* Staphylococcus aureus* all contribute to and interact with the diverse airway microbiome and the host immune system [58, 59]. It is very likely that immunosenescence plays a role in increasing susceptibility to respiratory infection in the elderly population. This is likely facilitated by an impaired mucosal barrier, reduced mucociliary clearance, and blunted airway immune and inflammatory responses on exposure to potentially pathogenic microorganisms [60]. Evidence of increased pulmonary “inflamm-aging” in the absence of infection has also been documented. For instance, increased CXCL10, an immune activation marker, and CD163 and increased CCL2 secretion, a macrophage recruiter, have all been described [61]. An alternate mechanism predisposing to infection in the elderly is reduced TLR function. There are significant impairments to TLR1 responses when measured in older individuals as compared to younger adults. TLR4 expression, critical for the response to* Streptococcus pneumoniae*, is also diminished among the elderly and during infection [62].

The elderly are highly susceptible to the influenza virus but respond poorly to vaccination [63]. Vaccinations can help to develop long term antibodies; however the virus constantly changes its coating proteins to evade neutralising antibodies. There is an impetus to develop vaccines that will target cell-mediated immunity in order to make them more effective and much ongoing work in the field is occurring [64]. Aging has been shown to reduce CD8 T-cell diversity and the immune response against influenza viral infection in mice [65]. Aging also leads to decreases in the number of naive and memory T-cells [66]. The currently used pneumococcal polysaccharide vaccine confers only some protection against* S. pneumonia* and several research groups continue to investigate novel mechanisms in understanding CD4 T-cell responses to vaccination in order to develop superior vaccines for clinical use [66].

While it is clear that the elderly remain at significant risks of pneumococcal infection and that immunosenescence plays a key role, influenza predisposes to secondary pneumococcal disease especially in this age group. This is subsequently further compounded by secondary inflammation and immune dysfunction [54]. A better understanding of the basic mechanisms that contribute to such increased risks of pulmonary infection is important to allow for the development of preventative methods and improved therapeutics in this particularly vulnerable cohort.

### 4.3. Chronic Obstructive Pulmonary Disease (COPD)

COPD affects over two hundred million individuals worldwide and represents a significant healthcare burden from both clinical and financial perspectives [67]. COPD occurs as a result of an increased inflammatory response incited by cigarette smoking or less likely environmental insult; however aging also represents an important contributing mechanism. This is because of altered anatomical lung structure and diminished innate immune function [67]. Aging in itself is associated with an increased incidence of chronic disease and COPD occurrence increases with age [68].

There is evidence that both aging and COPD share several common pathways and mechanisms. The innate immune system is suppressed in smokers and in individuals with COPD and there is an increase in susceptibility to infections and cancers [69]. Aging is associated with decreased epithelial barrier function [70], abnormalities in both cilia structure and function [71], and reduced production of antimicrobial and anti-inflammatory peptides produced by epithelial cells including SLPI [72]. Both COPD and aging are associated with an increased number of phagocytes including monocytes, macrophages, and neutrophils. Additionally, there is a reduction in host defense mechanisms including macrophage phagocytosis, ineffective chemotaxis, decreased bactericidal function of neutrophils, and altered capability of dendritic and natural killer cells [69]. “Inflamm-aging” often takes place and is associated with immunosuppression and low grade inflammation [69]. When secondary pulmonary infection occurs as a result of impaired host response, secondary inflammation develops [73].

During the natural aging process of healthy individuals, the lung undergoes a progressive decrease in lung function [74]. Studies have demonstrated that cigarette smoking accelerates the rate of lung function decline, suggesting that smoking results in premature aging of the lung [74]. Immunosenescence causes telomere shortening in leukocytes in COPD patients independent of the smoking status and causes increasing inflammatory markers, suggesting that certain disease specific factors may in fact be responsible for the premature aging of immune cells [75]. Evidence suggests that preceding lung function declines; smoking accelerates aging of the small airway epithelium by dysregulation of age-related gene expression and enhanced telomere erosion [76]. In support of this concept,* in vivo* studies have shown that alveolar epithelial cells from COPD smokers have increased numbers of senescent cells relative to healthy controls [77]. New hypotheses into mechanisms of COPD have drawn links between the natural aging lung and COPD smokers' lung and the role of increasing oxidative stress and apoptosis in emphysematous lungs [78].

### 4.4. Pulmonary Fibrosis

Several of the affected cellular and molecular mechanisms associated with the aging process are implicated in idiopathic pulmonary fibrosis (IPF). Patients with IPF also demonstrate increased markers of oxidative stress both within the airway and systemically [79]. In addition, evidence of an altered glutathione redox system has been described in the airway with reduced glutathione concentrations contained in alveolar lining fluid [80].

Telomeres are critically important chromosomal regions that ensure chromosome stability. They shorten naturally at the end of DNA replication but are also highly susceptible to oxidative stress and chronic inflammation. Such environmental insults cause intrinsic changes and shortening of the telomere region predisposing to shorter cell survival [81]. Telomere shortening is also a natural phenomenon observed during the aging process [82]. In patients with IPF, shortened telomeres in lung epithelia and peripheral blood cells have been described [83]. Where such telomeres reach a critical length, programmed cell arrest (senescence) and apoptosis occur. Abnormal cellular senescence is demonstrated in patients with IPF, particularly from bone marrow derived stem cells such as fibrocytes. Fibrocytes have been shown to traffic into the lungs in response to CXCL12 and to contribute to IPF pathogenesis [84]. Additionally, high levels of circulating fibrocytes have been shown to herald a poor prognosis in IPF [85]. A chronic background inflammatory state occurs in IPF that compares with immunosenescence associated “inflamm-aging.” Persistently low levels of IL-6 and TNF-*α* are observed with aging, whilst, in IPF, mildly elevated IL-8, IL-6, and CCL2 are detected [86].

Collagen and elastin are the major proteins making up the extracellular matrix (ECM) that forms the framework of the alveolar structure. Composition of the ECM changes during the aging process and subsequently contributes to age-related physiological declines in lung function [87]. Fibronectin expression increases in both clinical and experimental models of fibrosis and also with age. In injured lungs and particularly during the early phases of active repair, fibronectin production increases dramatically, and this increase occurs concurrent with fibroblast proliferation [68]. TGF-*β* is an important regulator of fibronectin and upregulates fibroblast proliferation [88]. It has been associated with the aging process and represents an important candidate for further study and potentially therapeutic manipulation. Recent evidence suggests that NADPH oxidase 4 is essential for TGF-*β*-induced differentiation of fibroblasts to myofibroblasts* in vitro* and for bleomycin-induced pulmonary fibrosis* in vivo* [89]. Senescent mouse lungs, following bleomycin-induced lung injury, recruit and maintain more fibroblasts, due partly to a loss in Thy1 expression in older mice, which in turn is under the influence of profibrotic cytokines such as TGF-*β*1. These can further undergo differentiation and further fibroblast production [90].

### 4.5. Autoimmune Disease, Vasculitis, and Other Respiratory Diseases

The elderly have a higher rate of autoimmunity but lower prevalence of autoimmune disease. The explanation for this is uncertain; however, it is postulated to be due to the increased expansion of peripheral regulatory T-cells. Autoimmunity may increase the affinity of T-cells to self-antigens or latent viruses promoting an autoimmune process [91]. Older adults have been shown to possess increased amounts of circulating autoantibodies due to the increased amount of tissue and cell damage coupled with apoptosis [92]. Importantly however higher levels of autoimmunity do not equate with increased autoimmune disease [93]. Thymic T-regulatory cells (Tregs) increase autoimmunity and reduce the CD4 and CD8 response which in turn increases susceptibility to infection and cancers. Recurrent bacterial and viral infections stimulate the release of proinflammatory cytokines which in turn are further expanded by activation of Tregs. Treg expansion is associated with T-helper 17 (Th17) cells and the persistence of chronic inflammation, a phenomenon that occurs during the physiological aging process [94].

The improved management of chronic respiratory disease results in increased longevity. Greater numbers of individuals are living longer and subsequently develop end-stage lung disease and its associated complications. This requires newer and advanced treatments including lung transplantation. Much of the available data however originates from renal transplant studies where it has been found that advanced donor and recipient age profiles are risk factors for poorer outcome. Elderly organ transplant recipients have dysfunctional alloimmune responses with an increased risk of chronic allograft failure and of acute rejection. With advancing age and immunosenescence, further challenges are posed by administering oral immunosuppression including alterations in their pharmacodynamics, pharmacokinetics, and compromised protein binding which all contribute to further illness and potential rejection in addition to other previously described age-associated factors [95]. In addition to immunosuppressive drug complexities, risks of infection and posttransplant malignancies are also increased [96]. This older posttransplant cohort poses unique challenges as most available clinical trials do not address nor include such patients in their analysis. An increased antigenic burden is also described in the older adult explained by “inflamm-aging” and potentially persistent subclinical infection [97, 98]. The true role of immunosenescence in posttransplant elderly patients remains uncertain; however the effects of aging on alloimmune responses and organ quality require further study and investigation to improve survival rates, reduce incidence of rejection, and optimize quality of life [99].

Age-associated increases occur both in the incidence and prevalence of cancer, suggestive of an association between the aging process and cancer development [100]. This relationship is not well understood particularly in the context of lung cancer; however, aging does promote cellular and molecular damage by previously described mechanisms and hence can promote cancer development. Cellular damage induced by either free radicals or viruses renders oncogenes more active and tumor suppressor genes inactive [101]. Certain immune-related adaptations occurring with age contribute specifically to cancer development. For example, under normal circumstances, the immune stimulation of T-cells by dendritic cells is critical for their activation and this is altered during aging. Furthermore, TLR signaling is also less effective with age resulting in aberrant responses and phagocytic dysfunction [102]. Ineffective neutrophil and macrophage function also contribute to the development and progression of tumors in the elderly [103].

## 5. Immunosenescence, the Airway Microbiome, and Effects on Pulmonary Disease

The emergence of the human “airway microbiome” has conferred unique challenges particularly for chronic inflammatory airway diseases such as asthma [104]. Organism numbers that have been detected within the microbiome are far greater than numbers of host cells with the amounts of microbial antigens being even greater. All such antigens can potentially interact with the host immune system and therefore the microbiome has relevance when considering physiological immunosenescence [105]. Because of this interaction between the microbiome and the host immune system within the airway, loss of immune function with age will likely have implications for the onset and progression of a variety of chronic pulmonary diseases particularly those associated with aging [106].

The interrelationship between host and microbiome particularly from an immunological context is in its infancy and publications continue to emerge associating it with pulmonary disease but data directly linking it with an aging immune system is lacking [106–108]. The described microbiome has continually been evolving and a fungal “mycobiome” has emerged [109]. Thus far, most of the identified microbiome is bacterial and able to maintain immune homeostasis. Microbes therefore importantly possess roles in both healthy and diseased states making understanding their role, interactions, and association with the immune system essential for the development of improved care and therapeutics [110]. The advent of molecular based microbiology such as 16S rRNA sequencing and metagenomics has transformed our practice of airway microbiology assessment [111]. Key factors that play a role in both healthy and diseased states include microbial species richness, community evenness, and diversity; however it is important to outline that no direct causation with a diseased state has been definitively established to date.

### 5.1. One Mucosal Hypothesis

We do not yet know if variation observed between individuals and their airway microbiomes mediates inflammation and subsequently confers damaging airway change directly or indirectly. This may be accounted for by interindividual systemic differences in immune function. One proposed concept is that of a “common mucosal system” where variations in gut microbiome development in early life serve to dictate systemic immune changes in later life. This would affect the airway if both the lung and gastrointestinal tract were part of the same continual mucosal spectrum. How would this in later life link to the physiological process of immunosenescence and the onset of pulmonary disease? Such questions coupled with airway exposure to the environment directly through respiration and the multiple medications used over a lifetime reveal what is likely a complex relationship where we still have much to learn.

### 5.2. The Gut and Pulmonary Microbiome

Age-associated change in gut microbe concentration fosters an imbalance* in vivo* that affects core physiological processes such as immunosenescence and “inflamm-aging.” First described as a broad association with disease, data on the gastrointestinal (GI) microbiome has driven the microbiome field over the last decade [112]. Understanding of the pulmonary microbiome was delayed not because of incorrect notions of a “sterile airway” but additionally because of difficulties in sampling the bronchus and preventing concurrent oropharyngeal contamination. It is now clearly accepted that variations in bacterial abundance, content, and structure occur in chronic inflammatory airway diseases such as cystic fibrosis (CF) [113], COPD [108], and asthma [104, 114].

The first study to describe disordered bacterial airway communities in asthma and COPD was performed by Hilty et al. [115]. This revealed that members of the Proteobacteria phylum (in particular* Haemophilus*) were prevalent in greater amounts in patients with COPD and asthma. Conversely, members of the Bacteroides phylum (such as* Prevotella*) were dominant in healthy subjects [115]. Critically, this work included both upper and lower airway samples separately and revealed that a particular microbiome may be characteristic of certain airway pathology. Further study replicated the early work but additionally assessed severity of airway hyperresponsiveness with bacterial diversity in asthmatic airways. Particular taxa associated with the findings included Proteobacteria, Pseudomonadaceae, Enterobacteriaceae, Burkholderiaceae, and Neisseriaceae [116]. Later assessment of sputum from steroid naive asthmatics confirmed more bacterial diversity and higher proportions of Proteobacteria [117].

While such emerging evidence illustrates an important role for the microbiome in asthma, these works do not address late-onset disease or any association to physiological processes such as aging or immunosenescence. Important invertebrate data has identified the key genes that control the aging process and modulate “healthy aging.” These include the IGF-1 signalling pathway, target of rapamycin (TOR) and AMP-activated protein kinase (AMPK). The microbiome can be influenced by these pathways and vice versa through interspecies signalling, metabolite production, nutrient deprivation, and host metabolic remodelling. The microbes interact with host transcriptional pathways and regulate across both species and their host by employing RNA and microRNA signalling mechanisms [118, 119]. It is yet uncertain how such animal data will advance our understanding of the aging process. Despite this, the very existence and interaction of the airway microbiome with the host immune system suggest that the effects of immunosenescence and inflamm-aging in the context of chronic inflammatory respiratory disease need to be examined more closely [104]. Factors linked to aging such as immune and inflammatory change combined with the lifelong impact of antimicrobial, allergic, and infective exposures place the microbiome found in the elderly likely somewhat different to that in the younger or healthy state.

## 6. Conclusion

The shift in global demographics as a consequence of increased life expectancies has given greater clinical and research focus to the physiological process of aging and its impact on chronic disease. The occurrence of respiratory illness increases with age and is exacerbated by immunosenescence and its associated inflammatory state. Influencing both innate and adaptive components of the immune system, immunosenescence shapes the clinical phenotype observed in many chronic respiratory diseases including asthma, COPD, and pulmonary fibrosis. This importantly differs from the same disease observed in younger cohorts. Age-related change in immunity additionally predisposes the elderly to pulmonary infection such as influenza and pneumococcus while a poorer vaccine response contributes to poorer outcomes. The emerging recognition of the gut and respiratory microbiome and their roles in disease pathogenesis poses further challenges for clinicians and researchers who need to understand the implications of such microorganisms in the context of an aging immune system. Further work is clearly necessary and requires investment to appreciate the physiological changes that occur with age and critically their impact on pulmonary disease which remains a key contributor to morbidity and mortality in older patients.

## References

[1] Vincent G. K., Velkoff V. A. (2010). *The Next Four Decades the Older Population in the United States: 2010–2050. Population Estimates and Projections*.

[2] United Nations (2013). *World Population Prospects: The 2012 Revision, Key Findings and Advance Tables*.

[3] Kaplan V., Angus D. C., Griffin M. F., Clermont G., Scott Watson R., Linde-Zwirble W. T. (2002). Hospitalized community-acquired pneumonia in the elderly: age- and sex-related patterns of care and outcome in the United States. *American Journal of Respiratory and Critical Care Medicine*.

[4] Ortiz J. R., Neuzil K. M., Rue T. C. (2013). Population-based incidence estimates of influenza-associated respiratory failure hospitalizations, 2003 to 2009. *The American Journal of Respiratory and Critical Care Medicine*.

[5] Tsai C.-L., Lee W.-Y., Hanania N. A., Camargo C. A. (2012). Age-related differences in clinical outcomes for acute asthma in the United States, 2006–2008. *The Journal of Allergy and Clinical Immunology*.

[6] Chotirmall S. H., Watts M., Branagan P., Donegan C. F., Moore A., McElvaney N. G. (2009). Diagnosis and management of asthma in older adults. *Journal of the American Geriatrics Society*.

[7] Al-Alawi M., Hassan T., Chotirmall S. H. (2014). Advances in the diagnosis and management of asthma in older adults. *The American Journal of Medicine*.

[8] Finch C. E., Ruvkun G. (2001). The genetics of aging. *Annual Review of Genomics and Human Genetics*.

[9] López-Otín C., Blasco M. A., Partridge L., Serrano M., Kroemer G. (2013). The hallmarks of aging. *Cell*.

[10] Thannickal V. J., Murthy M., Balch W. E. (2015). Blue journal conference. Aging and susceptibility to lung disease. *American Journal of Respiratory and Critical Care Medicine*.

[11] Aspinall R., Del Giudice G., Effros R. B., Grubeck-Loebenstein B., Sambhara S. (2007). Challenges for vaccination in the elderly. *Immunity & Ageing*.

[12] Gavazzi G., Krause K. H. (2002). Ageing and infection. *The Lancet Infectious Diseases*.

[13] Linton P. J., Dorshkind K. (2004). Age-related changes in lymphocyte development and function. *Nature Immunology*.

[14] Pawelec G., Akbar A., Beverley P. (2010). Immunosenescence and Cytomegalovirus: where do we stand after a decade?. *Immunity & Ageing*.

[15] Fulop T., le Page A., Fortin C., Witkowski J. M., Dupuis G., Larbi A. (2014). Cellular signaling in the aging immune system. *Current Opinion in Immunology*.

[16] Castelo-Branco C., Soveral I. (2014). The immune system and aging: a review. *Gynecological Endocrinology*.

[17] Franceschi C., Bonafè M., Valensin S. (2000). Inflamm-aging. An evolutionary perspective on immunosenescence. *Annals of the New York Academy of Sciences*.

[18] De la Fuente M., Miquel J. (2009). An update of the oxidation-inflammation theory of aging: the involvement of the immune system in oxi-inflamm-aging. *Current Pharmaceutical Design*.

[19] de Martinis M., Franceschi C., Monti D., Ginaldi L. (2005). Inflamm-ageing and lifelong antigenic load as major determinants of ageing rate and longevity. *FEBS Letters*.

[20] Bulati M., Pellicanò M., Vasto S., Colonna-Romano G. (2008). Understanding ageing: Biomedical and bioengineering approaches, the immunologic view. *Immunity and Ageing*.

[21] Alvarado C., Álvarez P., Puerto M., Gausserès N., Jiménez L., de la Fuente M. (2006). Dietary supplementation with antioxidants improves functions and decreases oxidative stress of leukocytes from prematurely aging mice. *Nutrition*.

[22] Wang C. Q., Udupa K. B., Xiao H., Lipschitz D. A. (1995). Effect of age on marrow macrophage number and function. *Aging: Clinical and Experimental Research*.

[23] Tsuboi I., Morimoto K., Hirabayashi Y. (2004). Senescent B lymphopoiesis is balanced in suppressive homeostasis: decrease in interleukin-7 and transforming growth factor-*β* levels in stromal cells of senescence-accelerated mice. *Experimental Biology and Medicine*.

[24] Gruver A. L., Hudson L. L., Sempowski G. D. (2007). Immunosenescence of ageing. *Journal of Pathology*.

[25] Whisler R. L., Grants I. S. (1993). Age-related alterations in the activation and expression of phosphotyrosine kinases and protein kinase C (PKC) among human B cells. *Mechanisms of Ageing and Development*.

[26] Macallan D. C., Wallace D. L., Zhang V. (2005). B-cell kinetics in humans: rapid turnover of peripheral blood memory cells. *Blood*.

[27] Haynes L., Eaton S. M., Burns E. M., Randall T. D., Swain S. L. (2005). Newly generated CD4 T cells in aged animals do not exhibit age-related defects in response to antigen. *The Journal of Experimental Medicine*.

[28] Ongrádi J., Kövesdi V. (2010). Factors that may impact on immunosenescence: an appraisal. *Immunity & Ageing*.

[29] Amelink M., de Nijs S. B., de Groot J. C. (2013). Three phenotypes of adult-onset asthma. *Allergy*.

[30] Amelink M., de Groot J. C., de Nijs S. B. (2013). Severe adult-onset asthma: a distinct phenotype. *The Journal of Allergy and Clinical Immunology*.

[31] Sakuishi K., Oki S., Araki M., Porcelli S. A., Miyake S., Yamamura T. (2007). Invariant NKT cells biased for IL-5 production act as crucial regulators of inflammation. *Journal of Immunology*.

[32] Johnson S. A., Rozzo S. J., Cambier J. C. (2002). Aging-dependent exclusion of antigen-inexperienced cells from the peripheral B cell repertoire. *Journal of Immunology*.

[33] Gill J., Malin M., Sutherland J., Gray D., Hollander G., Boyd R. (2003). Thymic generation and regeneration. *Immunological Reviews*.

[34] Mathur S. K., Schwantes E. A., Jarjour N. N., Busse W. W. (2008). Age-related changes in eosinophil function in human subjects. *Chest*.

[35] Olin A.-C., Rosengren A., Thelle D. S., Lissner L., Bake B., Torén K. (2006). Height, age, and atopy are associated with fraction of exhaled nitric oxide in a large adult general population sample. *Chest*.

[36] Aaron S. D., Angel J. B., Lunau M. (2001). Granulocyte inflammatory markers and airway infection during acute exacerbation of chronic obstructive pulmonary disease. *American Journal of Respiratory and Critical Care Medicine*.

[37] Thomas R. A., Green R. H., Brightling C. E. (2004). The influence of age on induced sputum differential cell counts in normal subjects. *Chest*.

[38] Mathur S. K. (2010). Allergy and asthma in the elderly. *Seminars in Respiratory and Critical Care Medicine*.

[39] Cardona V., Guilarte M., Luengo O., Labrador-Horrillo M., Sala-Cunill A., Garriga T. (2011). Allergic diseases in elderly. *Clinical and Translational Allergy*.

[40] Mocchegiani E., Malavolta M., Costarelli L. (2010). Zinc, metallothioneins and immunosenescence. *Proceedings of the Nutrition Society*.

[41] Pietschmann P., Woloszczuk W., Pietschmann H. (1990). Increased serum osteocalcin levels in elderly females with vitamin D deficiency. *Experimental and Clinical Endocrinology*.

[42] Scichilone N., Callari A., Augugliaro G., Marchese M., Togias A., Bellia V. (2011). The impact of age on prevalence of positive skin prick tests and specific IgE tests. *Respiratory Medicine*.

[43] Pelaia G., Vatrella A., Busceti M. T. (2015). Cellular mechanisms underlying eosinophilic and neutrophilic airway inflammation in asthma. *Mediators of Inflammation*.

[44] Hellings P. W., Kasran A., Liu Z. (2003). Interleukin-17 orchestrates the granulocyte influx into airways after allergen inhalation in a mouse model of allergic asthma. *The American Journal of Respiratory Cell and Molecular Biology*.

[45] Oda N., Canelos P. B., Essayan D. M., Plunkett B. A., Myers A. C., Huang S.-K. (2005). Interleukin-17F induces pulmonary neutrophilia and amplifies antigen-induced allergic response. *American Journal of Respiratory and Critical Care Medicine*.

[46] Wakashin H., Hirose K., Maezawa Y. (2008). IL-23 and Th17 cells enhance Th2-cell-mediated eosinophilic airway inflammation in mice. *American Journal of Respiratory and Critical Care Medicine*.

[47] Finkelman F. D., Hogan S. P., Khurana Hershey G. K., Rothenberg M. E., Wills-Karp M. (2010). Importance of cytokines in murine allergic airway disease and human asthma. *Journal of Immunology*.

[48] Molet S., Hamid Q., Davoine F. (2001). IL-17 is increased in asthmatic airways and induces human bronchial fibroblasts to produce cytokines. *The Journal of Allergy and Clinical Immunology*.

[49] Happel K. I., Dubin P. J., Zheng M. (2005). Divergent roles of IL-23 and IL-12 in host defense against *Klebsiella pneumoniae*. *The Journal of Experimental Medicine*.

[50] Stout-Delgado H. W., Du W., Shirali A. C., Booth C. J., Goldstein D. R. (2009). Aging promotes neutrophil-induced mortality by augmenting IL-17 production during viral infection. *Cell Host & Microbe*.

[51] High K. P., Prasad R., Marion C. R., Schurig G. G., Boyle S. M., Sriranganathan N. (2007). Outcome and immune responses after *Brucella abortus* infection in young adult and aged mice. *Biogerontology*.

[52] Loeb M., McGeer A., McArthur M., Walter S., Simor A. E. (1999). Risk factors for pneumonia and other lower respiratory tract infections in elderly residents of long-term care facilities. *Archives of Internal Medicine*.

[53] Kikawada M., Iwamoto T., Takasaki M. (2005). Aspiration and infection in the elderly: epidemiology, diagnosis and management. *Drugs and Aging*.

[54] Van der Sluijs K. F., Van der Poll T., Lutter R., Juffermans N. P., Schultz M. J. (2010). Bench-to-bedside review: bacterial pneumonia with influenza—pathogenesis and clinical implications. *Critical Care*.

[55] Regev-Yochay G., Raz M., Dagan R. (2004). Nasopharyngeal carriage of *Streptococcus pneumoniae* by adults and children in community and family settings. *Clinical Infectious Diseases*.

[56] Suzuki N., Yuyama M., Maeda S., Ogawa H., Mashiko K., Kiyoura Y. (2006). Genotypic identification of presumptive *Streptococcus pneumoniae* by PCR using four genes highly specific for *S. pneumoniae*. *Journal of Medical Microbiology*.

[57] Krone C. L., van de Groep K., Trzciński K., Sanders E. A. M., Bogaert D. (2014). Immunosenescence and pneumococcal disease: an imbalance in host-pathogen interactions. *The Lancet Respiratory Medicine*.

[58] Bogaert D., Keijser B., Huse S. (2011). Variability and diversity of nasopharyngeal microbiota in children: a metagenomic analysis. *PLoS ONE*.

[59] Charlson E. S., Bittinger K., Haas A. R. (2011). Topographical continuity of bacterial populations in the healthy human respiratory tract. *The American Journal of Respiratory and Critical Care Medicine*.

[60] Incalzi R. A., Maini C. L., Fuso L., Giordana A., Carbonin P. U., Galli G. (1989). Effects of aging on mucociliary clearance. *Comprehensive Gerontology Section A: Clinical and Laboratory Sciences*.

[61] Seidler S., Zimmermann H. W., Bartneck M., Trautwein C., Tacke F. (2010). Age-dependent alterations of monocyte subsets and monocyte-related chemokine pathways in healthy adults. *BMC Immunology*.

[62] van Duin D., Mohanty S., Thomas V. (2007). Age-associated defect in human TLR-1/2 function. *Journal of Immunology*.

[63] Webster R. G. (2000). Immunity to influenza in the elderly. *Vaccine*.

[64] McElhaney J. E., Xie D., Hager W. D. (2006). T cell responses are better correlates of vaccine protection in the elderly. *Journal of Immunology*.

[65] Toapanta F. R., Ross T. M. (2009). Impaired immune responses in the lungs of aged mice following influenza infection. *Respiratory Research*.

[66] Lanzer K. G., Johnson L. L., Woodland D. L., Blackman M. A. (2014). Impact of ageing on the response and repertoire of influenza virus-specific CD4 T cells. *Immunity & Ageing*.

[67] Decramer M., Janssens W., Miravitlles M. (2012). Chronic obstructive pulmonary disease. *The Lancet*.

[68] Faner R., Rojas M., MacNee W., Agustí A. (2012). Abnormal lung aging in chronic obstructive pulmonary disease and idiopathic pulmonary fibrosis. *American Journal of Respiratory and Critical Care Medicine*.

[69] Fulop T., Larbi A., Kotb R., de Angelis F., Pawelec G. (2011). Aging, immunity, and cancer. *Discovery medicine*.

[70] Ghadially R., Brown B. E., Sequeira-Martin S. M., Feingold K. R., Elias P. M. (1995). The aged epidermal permeability barrier. Structural, functional, and lipid biochemical abnormalities in humans and a senescent murine model. *The Journal of Clinical Investigation*.

[71] Ho J. C., Chan K. N., Hu W. H. (2001). The effect of aging on nasal mucociliary clearance, beat frequency, and ultrastructure of respiratory cilia. *American Journal of Respiratory and Critical Care Medicine*.

[72] Shugars D. C., Watkins C. A., Cowen H. J. (2001). Salivary concentration of secretory leukocyte protease inhibitor, an antimicrobial protein, is decreased with advanced age. *Gerontology*.

[73] Sethi S., Murphy T. F. (2008). Infection in the pathogenesis and course of chronic obstructive pulmonary disease. *The New England Journal of Medicine*.

[74] Kerstjens H. A. M., Rijcken B., Scheuten J. P., Postma D. S. (1997). Decline of FEV1 by age and smoking status: facts, figures, and fallacies. *Thorax*.

[75] Morlá M., Busquets X., Pons J., Sauleda J., MacNee W., Agustí A. G. N. (2006). Telomere shortening in smokers with and without COPD. *The European Respiratory Journal*.

[76] Walters M. S., De B. P., Salit J. (2014). Smoking accelerates aging of the small airway epithelium. *Respiratory Research*.

[77] Tsuji T., Aoshiba K., Nagai A. (2006). Alveolar cell senescence in patients with pulmonary emphysema. *American Journal of Respiratory and Critical Care Medicine*.

[78] MacNee W., Tuder R. M. (2009). New paradigms in the pathogenesis of chronic obstructive pulmonary disease I. *Proceedings of the American Thoracic Society*.

[79] Rahman I., Skwarska E., Henry M. (1999). Systemic and pulmonary oxidative stress in idiopathic pulmonary fibrosis. *Free Radical Biology and Medicine*.

[80] Beeh K. M., Beier J., Haas I. C., Kornmann O., Micke P., Buhl R. (2002). Glutathione deficiency of the lower respiratory tract in patients with idiopathic pulmonary fibrosis. *European Respiratory Journal*.

[81] Saretzki G., Von Zglinicki T. (2002). Replicative aging, telomeres, and oxidative stress. *Annals of the New York Academy of Sciences*.

[82] Kaszubowska L. (2008). Telomere shortening and ageing of the immune system. *Journal of Physiology and Pharmacology*.

[83] Borie R., Crestani B., Bichat H. (2009). Prevalence of telomere shortening in familial and sporadic pulmonary fibrosis is increased in men. *The American Journal of Respiratory and Critical Care Medicine*.

[84] Moore B. B. (2009). Fibrocytes as potential biomarkers in idiopathic pulmonary fibrosis. *American Journal of Respiratory and Critical Care Medicine*.

[85] Moeller A., Gilpin S. E., Ask K. (2009). Circulating fibrocytes are an indicator of poor prognosis in idiopathic pulmonary fibrosis. *American Journal of Respiratory and Critical Care Medicine*.

[86] Harari S., Caminati A. (2010). IPF: new insight on pathogenesis and treatment. *Allergy*.

[87] Takayanagi N., Kagiyama N., Ishiguro T., Tokunaga D., Sugita Y. (2010). Etiology and outcome of community-acquired lung abscess. *Respiration*.

[88] Fernandez I. E., Eickelberg O. (2012). The impact of TGF-*β* on lung fibrosis: From targeting to biomarkers. *Proceedings of the American Thoracic Society*.

[89] Jiang F., Liu G.-S., Dusting G. J., Chan E. C. (2014). NADPH oxidase-dependent redox signaling in TGF-*β*-mediated fibrotic responses. *Redox Biology*.

[90] Sueblinvong V., Neveu W. A., Neujahr D. C. (2014). Aging promotes pro-fibrotic matrix production and increases fibrocyte recruitment during acute lung injury. *Advances in Bioscience and Biotechnology*.

[91] Vadasz Z., Haj T., Kessel A., Toubi E. (2013). Age-related autoimmunity. *BMC Medicine*.

[92] Candore G., Di Lorenzo G., Mansueto P. (1997). Prevalence of organ-specific and non organ-specific autoantibodies in healthy centenarians. *Mechanisms of Ageing and Development*.

[93] Andersen-Ranberg K., Høier-Madsen M., Wiik A., Jeune B., Hegedüs L. (2004). High prevalence of autoantibodies among Danish centenarians. *Clinical and Experimental Immunology*.

[94] Sun L., Hurez V. J., Thibodeaux S. R. (2012). Aged regulatory T cells protect from autoimmune inflammation despite reduced STAT3 activation and decreased constraint of IL-17 producing T cells. *Aging Cell*.

[95] Danovitch G. M., Gill J., Bunnapradist S. (2007). Immunosuppression of the elderly kidney transplant recipient. *Transplantation*.

[96] Danpanich E., Kasiske B. L. (1999). Risk factors for cancer in renal transplant recipients. *Transplantation*.

[97] Franceschi C., Capri M., Monti D. (2007). Inflammaging and anti-inflammaging: a systemic perspective on aging and longevity emerged from studies in humans. *Mechanisms of Ageing and Development*.

[98] Ershler W. B., Keller E. T. (2000). Age-associated increased interleukin-6 gene expression, late-life diseases, and frailty. *Annual Review of Medicine*.

[99] Heinbokel T., Elkhal A., Liu G., Edtinger K., Tullius S. G. (2013). Immunosenescence and organ transplantation. *Transplantation Reviews*.

[100] Extermann M. (2013). *Cancer and Aging. From Bench to Clinics*.

[101] Davalos A. R., Coppe J.-P., Campisi J., Desprez P.-Y. (2010). Senescent cells as a source of inflammatory factors for tumor progression. *Cancer and Metastasis Reviews*.

[102] Balistreri C. R., Colonna-Romano G., Lio D., Candore G., Caruso C. (2009). TLR4 polymorphisms and ageing: implications for the pathophysiology of age-related diseases. *Journal of Clinical Immunology*.

[103] Fortin C. F., McDonald P. P., Lesur O., Fülöp T. (2008). Aging and neutrophils: there is still much to do. *Rejuvenation Research*.

[104] Chotirmall S. H., Burke C. M. (2015). Aging and the microbiome: implications for asthma in the elderly?. *Expert Review of Respiratory Medicine*.

[105] Human Microbiome Project Consortium (2012). Structure, function and diversity of the healthy human microbiome. *Nature*.

[106] Segal L. N., Rom W. N., Weiden M. D. (2014). Lung microbiome for clinicians. New discoveries about bugs in healthy and diseased lungs. *Annals of the American Thoracic Society*.

[107] Sethi S. (2014). Chronic obstructive pulmonary disease and infection. Disruption of the microbiome?. *Annals of the American Thoracic Society*.

[108] Huang Y. J., Sethi S., Murphy T., Nariya S., Boushey H. A., Lynch S. V. (2014). Airway microbiome dynamics in exacerbations of chronic obstructive pulmonary disease. *Journal of Clinical Microbiology*.

[109] Nguyen L. D., Viscogliosi E., Delhaes L. (2015). The lung mycobiome: an emerging field of the human respiratory microbiome. *Frontiers in Microbiology*.

[110] The Human Microbiome Project Consortium (2012). A framework for human microbiome research. *Nature*.

[111] Cox M. J., Cookson W. O. C. M., Moffatt M. F. (2013). Sequencing the human microbiome in health and disease. *Human Molecular Genetics*.

[112] Atarashi K., Tanoue T., Oshima K. (2013). T_reg_ induction by a rationally selected mixture of Clostridia strains from the human microbiota. *Nature*.

[113] Chmiel J. F., Aksamit T. R., Chotirmall S. H. (2014). Antibiotic management of lung infections in cystic fibrosis. I. The microbiome, methicillin-resistant *Staphylococcus aureus*, gram-negative bacteria, and multiple infections. *Annals of the American Thoracic Society*.

[114] Han M. K., Huang Y. J., LiPuma J. J. (2012). Significance of the microbiome in obstructive lung disease. *Thorax*.

[115] Hilty M., Burke C., Pedro H. (2010). Disordered microbial communities in asthmatic airways. *PLoS ONE*.

[116] Huang Y. J., Nelson C. E., Brodie E. L. (2011). Airway microbiota and bronchial hyperresponsiveness in patients with suboptimally controlled asthma. *Journal of Allergy and Clinical Immunology*.

[117] Marri P. R., Stern D. A., Wright A. L., Billheimer D., Martinez F. D. (2013). Asthma-associated differences in microbial composition of induced sputum. *The Journal of Allergy and Clinical Immunology*.

[118] Zhang R., Hou A. (2013). Host-microbe interactions in *Caenorhabditis elegans*. *ISRN Microbiology*.

[119] Heintz C., Mair W. (2014). You are what you host: microbiome modulation of the aging process. *Cell*.

